# Immunohistochemical Determination of p53 Protein Overexpression for Predicting p53 Gene Mutations in Hepatocellular Carcinoma: A Meta-Analysis

**DOI:** 10.1371/journal.pone.0159636

**Published:** 2016-07-18

**Authors:** Jiangbo Liu, Wei Li, Miao Deng, Dechun Liu, Qingyong Ma, Xiaoshan Feng

**Affiliations:** 1 Department of General Surgery, First Affiliated Hospital, College of Clinical Medicine, Henan University of Science and Technology, Luoyang, Henan, PR China; 2 Department of Hepatobiliary Surgery, First Affiliated Hospital of Xi’an Jiaotong University, Xi’an, Shaanxi, PR China; 3 Henan Key Laboratory of Cancer Epigenetics, Cancer Institute, First Affiliated Hospital, College of Clinical Medicine, Henan University of Science and Technology, Luoyang, Henan, PR China; University of North Carolina School of Medicine, UNITED STATES

## Abstract

**Background:**

Whether increased expression of the tumor suppressor protein p53 indicates a p53 gene mutation in hepatocellular carcinoma (HCC) remains unclear. We conducted a meta-analysis to determine whether p53 protein overexpression detected by immunohistochemistry (IHC) offers a diagnostic prediction for p53 gene mutations in HCC patients.

**Methods:**

Systematic literature searches were conducted with an end date of December 2015. A meta-analysis was performed to estimate the diagnostic accuracy of IHC-determined p53 protein overexpression in the prediction of p53 gene mutations in HCC. Sensitivity, subgroup, and publication bias analyses were also conducted.

**Results:**

Thirty-six studies were included in the meta-analysis. The results showed that the overall sensitivity and specificity for IHC-determined p53 overexpression in the diagnostic prediction of p53 mutations in HCC were 0.83 (95% CI: 0.80–0.86) and 0.74 (95% CI: 0.71–0.76), respectively. The summary positive likelihood ratio (PLR) and negative likelihood ratio (NLR) were 2.65 (95% CI: 2.21–3.18) and 0.36 (95% CI: 0.26–0.50), respectively. The diagnostic odds ratio (DOR) of IHC-determined p53 overexpression in predicting p53 mutations ranged from 0.56 to 105.00 (pooled, 9.77; 95% CI: 6.35–15.02), with significant heterogeneity between the included studies (*I*^2^ = 40.7%, *P* = 0.0067). Moreover, subgroup and sensitivity analyses did not alter the results of the meta-analysis. However, potential publication bias was present in the current meta-analysis.

**Conclusion:**

The upregulation of the tumor suppressor protein p53 was indeed linked to p53 gene mutations. IHC determination of p53 overexpression can predict p53 gene mutations in HCC patients.

## Introduction

Hepatocellular carcinoma (HCC) is one of the most prevalent cancers worldwide, and the cancer-related deaths due to this condition are increasing [1,2]. Therefore, elucidating the malignant biological features of HCC is critical for outcome prediction in patients with this disease. Mutations in the tumor suppressor gene p53 are the most common genetic changes in human malignancies. In HCC, the frequency of p53 gene mutations is as high as 50.0% (average 30.0%); therefore, analysis of this gene and its products is of practical importance [3,4]. Several studies have reported that alterations of the p53 gene are correlated with tumor differentiation, vascular invasion, and tumor stage in HCC [5–7]. Moreover, aberrations of the p53 gene have been shown to be prognostic indicators associated with recurrence-free survival and overall survival in HCC patients [3,8].

Wild-type p53 protein is responsible for cell cycle regulation and apoptosis following DNA damage, while mutant p53 protein shows a loss of function [8,9]. Mutational analysis using a variety of techniques, such as direct DNA sequencing, single-strand conformation polymorphism (SSCP) analysis followed by DNA sequencing, and other mutation assays, is the gold standard for the identification of p53 genetic alterations [8–11]. Generally, the transition from wild-type p53 to a mutant phenotype results in mutant p53 protein overexpression due to the resistance to murine double minute gene 2 (MDM2)-mediated degradation and subsequent abnormal stability of the mutant protein; therefore, immunohistochemistry (IHC) can be used to determine the expression and location of mutant p53 protein that has accumulated in the cell nuclei of cancer tissues [12,13]. IHC is an economic and convenient technology; thus, more clinical studies have adopted IHC to identify genetic alterations in the p53 gene rather than using mutational analysis [3]. However, it remains unclear whether a concordance exists between p53 protein overexpression and p53 gene mutations in HCC patients. As reported in a previously published meta-analysis, the association between p53 mutations and p53 overexpression in predicting shorter patient survival times in HCC suggested a correlation between p53 expression and p53 mutations [3]. However, several studies have found that p53 expression determined by IHC assays did not predict p53 mutations [14–16]. Moreover, the accuracy of IHC in measuring p53 protein overexpression for the prediction of p53 mutations in HCC is not clear.

To determine whether p53 protein overexpression is concordant with p53 gene mutations, we performed a diagnostic meta-analysis of relevant observational studies. We evaluated the ability of IHC assessment of p53 protein overexpression to predict p53 mutations identified by mutational analysis as a reference standard in HCC.

## Materials and Methods

### Literature search

A comprehensive literature search was conducted using the National Center for Biotechnology Information PubMed (MEDLINE) databases with an end date of December 2015 using the following search terms: (liver neoplasm or hepatocellular carcinoma or carcinoma, hepatocellular or HCC) and (tumor suppressor protein p53 or p53) and (immunohistochemistry or IHC or immunostaining or immunoassay or expression or overexpression or up-regulation) and (mutation or mutational analysis or DNA mutational analysis). References in the selected studies and review articles were also manually assessed.

### Study selection

Studies were required to meet the following inclusion criteria: (1) provided a confirmed diagnosis of HCC in humans; (2) explicitly reported the detection methods for p53 alterations, including IHC, the specific antibodies used to determine p53 protein overexpression and mutational assays, such as PCR-SSCP and/or DNA sequencing, or other specific approaches for identifying p53 gene mutations; (3) provided data on p53 expression and p53 mutations, with the prevalence of p53 mutations greater than 0%; and (4) written in English, German, or Chinese.

Two investigators (Jiang-Bo Liu and Wei Li) independently read the title and abstract of candidate studies, and irrelevant studies were excluded if they did not meet the inclusion criteria. Then, the two investigators analyzed the full texts of the selected studies and determined whether the studies should be included. If disagreements occurred, the two investigators conducted a discussion or recruited the third investigator (Miao Deng) until a consensus was reached. Additionally, if studies were found to employ overlapping populations after comprehensive evaluation, the one with the largest population or the newest study was usually included.

### Data extraction and quality assessment

Two investigators (Jiang-Bo Liu and Wei Li) independently extracted the data, which included the first author, publication year, recruitment period, geographic location, sample size, analytical method (protein/gene), and cut-off values/detected exons. Moreover, the diagnostic data, including the true positive (TP), false positive (FP), false negative (FN), and true negative (TN) values of IHC-determined p53 expression levels and p53 mutations identified by mutational analysis (as a reference standard), were extracted from the relevant articles. The revised version of the Quality Assessment of Diagnostic Accuracy Studies (QUADAS-2) tool, comprising 4 domains (11 items), was used to assess the quality of all included studies [17].

### Statistical analysis

The statistical software Meta-DiSc version 1.4 (XI Cochrane Colloquium, Barcelona, Spain) and Stata version 12 (Stata Corporation, College Station, TX, USA) were used in the meta-analysis. Accordingly, TP, TN, FP, and FN were retrieved from each article. The summary sensitivity (SEN), specificity (SPE), positive likelihood ratio (PLR), negative likelihood ratio (NLR), and diagnostic odds ratio (DOR) estimates with 95% confidence intervals (CIs) were analyzed using a random-effects model, and a bivariate summary receiver operating characteristic (SROC) curve was generated. The area under the SROC curve (AUC) represented an analytical summary of the test performance and illustrated the trade-off between SEN and SPE. The between-studies heterogeneity was evaluated with the *I*^2^ statistic (range 0% to 100%), and an *I*^2^ statistic index greater than 50% indicated substantial heterogeneity [18]. Sensitivity analyses were performed to explore possible heterogeneity, and the influence of individual studies on the meta-analytical results was assessed by applying the leave-one-out method. Deeks’ funnel plots were generated to explore potential publication bias, with *P*-values less than 0.1 indicating significance [19].

## Results

### Search results

**Fig 1:**
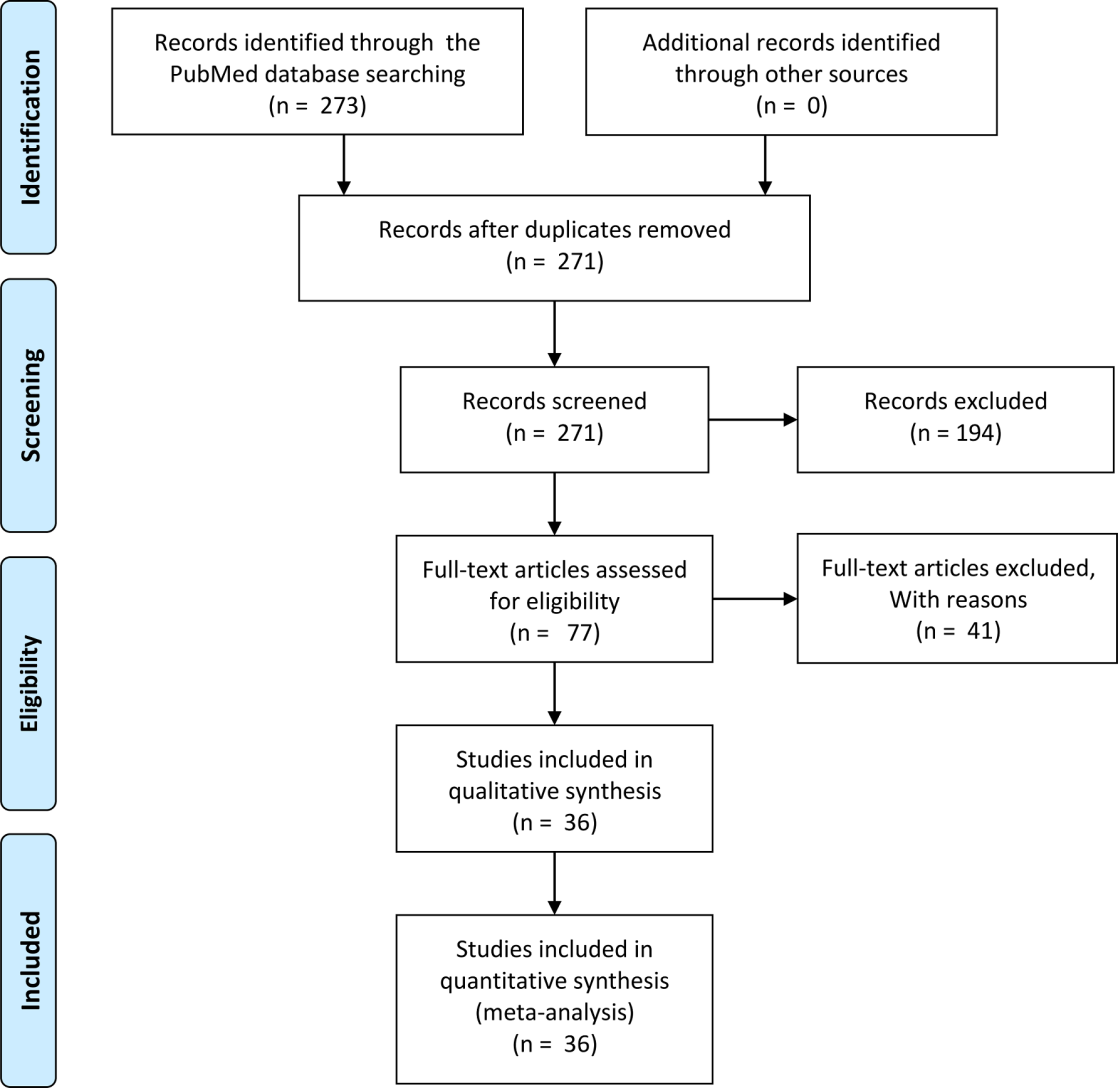
Flow chart of the selection process for the included studies.

An initial search retrieved 273 published studies. After a careful selection process, thirty-six relevant observational studies (34 in English [4,5,8–12,14–16,20–43] and 2 in Chinese [44,45]) were included in the meta-analysis. Fig 1 shows the literature screening process for the meta-analysis. The included studies had QUADAS-2 scores of 9 to 11 (median = 10).

### Characteristics of the included studies

The characteristics of each study are shown in Table 1. Of 36 studies, 23 were conducted in high-incidence areas (Asia and Africa) [4,5,8,10–12,23–29,31,34,35,38–40,42–45], and 13 were conducted in low-incidence areas (Europe and USA) [9,14–16,20–22,30,32,33,36,37,41]. The studies included 1,659 HCC patients with a mean sample size of 46 (range 8 to 397). Among the included studies, 584 cases of p53 gene mutations and 765 cases of p53 protein overexpression were found in HCC tissues, with an average mutation and overexpression prevalence of 35.2% (range 2.9% to 60.7%) and 46.1% (range 5.0% to 72.7%), respectively. Thirty-two studies described mutable sites of the p53 gene, reporting 822 mutations in 584 cases of HCC, while another four studies did not report specific sites. Of the 822 reported p53 mutations, the most frequently mutated sites were exons 5 and 7, accounting for 14.2% and 68.9% of the reported mutations, respectively, and codon 249 located in exon 7 had the highest mutation rate of 30.1% (248/822).

**Table 1:** The analytical results of correlations between p53 mutations and p53 overexpression.

Reference	Country	Potential mutagen	Sample size	TP	FP	FN	TN	IHC cut-off/Exon	Analytical method (antibody/gene)	QUADAS-2
An et al. 2001 [35]	Japan	HCV	11 of 41	1	7	0	3	NA/exons 5–8	IHC (DO-7)/PCR-DNA sequencing	11
Andersson et al. 1995 [33]	Denmark	alpha-particles	18 of 36	0	2	1	15	1%/exons 5,7,8	IHC (DO-7)/PCR-DGGE, DNA sequencing	9
Anzola et al. 2004 [15]	Spain	HCV, alcohol	117 from 78	4	23	8	82	10%/exons 4–8	IHC (DO-7)/PCR-SSCP, DNA sequencing	11
Boix-Ferrero et al. 1999 [20]	Spain	HCV, alcohol	70 of 129	1	13	1	55	10%/exons 5–8	IHC (Bp 53–11)/PCR-DNA sequencing	11
Bourdon et al. 1995 [14]	France	HBV	20	5	5	1	9	NA/exons 2–11	IHC (PAb1801)/PCR-DNA sequencing	10
Challen et al. 1992 [22]	UK	–*	19	1	0	1	17	10%/exons 5–8	IHC (–)/PCR-DNA sequencing	9
Chen et al. 2003 [43]	China	HBV	33	16	5	0	12	NA/exons 2–8	IHC (Santa)/PCR-DNA sequencing	10
Cheung et al. 2006 [4]	China	HBV	55	17	11	9	18	NA/exons 4–9	IHC (DO-7)/PCR-DNA sequencing	11
De Benedetti et al. 1996 [41]	USA	Contraceptive	10 of 11	1	2	0	7	1%/exons 4–9	IHC (–)/PCR-DNA sequencing	9
Greenblatt et al. 1997 [29]	China	HBV	16	1	5	2	8	1%/exons 4–8	IHC (CM-1)/PCR-DNA sequencing	9
Gross-Goupil et al. 2003 [16]	France	HBV, HCV	18	0	4	2	12	10%/exons 2–11	IHC (DO-7)/PCR-DNA sequencing	10
Hsia et al. 2000 [24]	China	–*	28	16	3	1	8	10%/exons 5–8	IHC (–)/PCR-DNA sequencing	9
Hsu et al. 1993 [26]	China	HBV, HCV	78 of 184	30	9	10	29	10%/exons 2–11	IHC (DO-7)/PCR-SSCP, DNA sequencing	9
Jablkowski et al. 2005 [9]	Poland	HBV	9 of 20	4	1	1	3	50%/exons 5–8	IHC (DO-7)/PCR-DNA sequencing	10
Kang et al. 1998 [39]	Korea	HBV	8 of 13	2	2	0	4	20%/exons 5–8	IHC (DO-7)/PCR-SSCP, DNA sequencing	9
Kubicka et al. 1995 [32]	Germany	HBV	20	1	0	1	18	30%/exons 5–8	IHC (PAb1801/PAb240) /PCR-DNA sequencing	9
Lee et al. 2002 [25]	Korea	HBV	36 from 34	6	9	1	20	5%/exons 4–10	IHC (BP53-12)/PCR-SSCP, DNA sequencing	10
Lunn et al. 1997 [42]	China	HBV, AFB1	105	22	13	7	63	5%/exons 5–9	IHC (DO-1/Ab-6)/PCR-SSCP, DNA sequencing	9
Luo et al. 2001 [45]	China	–	21	6	5	3	7	10%/exons 5–8	IHC (DO-7)/PCR-SSCP	10
Mitsumoto et al. 2004 [31]	Japan	HCV	50	13	1	8	28	10%/–	IHC (DO-7)/Yeast p53 Functional Assay, DNA sequencing	9
Mohamed et al. 2008 [5]	Egypt	HBV, HCV	30	7	9	4	10	10%/exons 5–8	IHC (DO-7)/PCR-SSCP, DNA sequencing	11
Okada et al. 2003 [27]	Japan	HCV	10 of 22	5	1	0	4	10%/exons 5–9	IHC (DO-7)/PCR-DNA sequencing	10
Peng et al. 1998 [23]	China	–	70	21	9	2	38	5%/exon 7	IHC (DO-7)/RFLP	9
Qi et al. 2015 [8]	China	HBV, AFB1	397	208	58	15	116	25%/exons 1–11	IHC (Abcam)/PCR-DNA sequencing	11
Qin et al. 1998 [38]	China	HBV	26 of 31	5	1	1	19	NA/exons 5–9	IHC (PAb1801/PAb240) /PCR-DNA sequencing	9
Rashid et al. 1999 [10]	China	HBV	24	10	3	2	9	NA/exons 2–9	IHC (DO-7)/PCR-DNA sequencing	11
Ryder et al. 1996 [37]	Germany	HBV, HCV	37 of 38	15	3	2	17	80%/exons 5–8	IHC (DO-1)/PCR-SSCP, DNA sequencing	10
Sanefuji et al. 2010 [34]	Japan	HCV	79 of 82	13	34	0	32	10%/exons 5–8	IHC (DO-7)/PCR-DNA sequencing	11
Shieh et al. 1993 [36]	USA	HBV, HCV	18	1	0	0	17	NA/exon 7	IHC (PAb1801)/PCR-DNA sequencing	9
Soini et al. 1996 [21]	Mexico	AFB1	14 of 21	2	4	1	7	1%/exon 7	IHC (CM-1)/PCR-DNA sequencing	9
Stern et al. 2001 [28]	China	HBV, AFB1	48 of 64	15	15	3	15	NA/exon 7	IHC (CM-1)/PCR-DNA sequencing	10
Szymańska et al. 2004 [11]	Gambia	HBV	28 of 29	9	4	5	10	10%/exons 5–8	IHC (CM-1)/PCR-RFLP, DNA sequencing/SOMA	9
Volkmann et al. 2001 [30]	Germany	HBV, HCV	39	8	3	3	25	10%/exons 5–9	IHC (DO-1)/PCR-SSCP, DNA sequencing	10
Zekri et al. 2006 [40]	Egypt	HCV	25	7	6	3	9	10%/exons 5–8	IHC (DO-7)/PCR-SSCP, DNA sequencing	9
Zhang et al. 2006 [12]	China	HBV, AFB1	40	9	5	2	24	5%/exons 5–8	IHC (DO-7)/PCR-DNA sequencing	10
Zhou et al. 1997 [44]	China	HBV	32	2	6	0	24	NA/exon 7	IHC (DO-1/Ab-6)/PCR-RFLP	9

HBV/HCV: hepatitis B/C virus; AFB1: aflatoxin B1; IHC: immunohistochemistry; PCR: polymerase chain reaction; SSCP: single-strand conformation polymorphism; RFLP: restriction fragment length polymorphism; SOMA: short oligonucleotide mass spectrometry analysis; DGGE: denaturing gradient gel electrophoresis; QUADAS, Quality Assessment of Diagnostic accuracy studies.

### Diagnostic accuracy analysis

**Fig 2:**
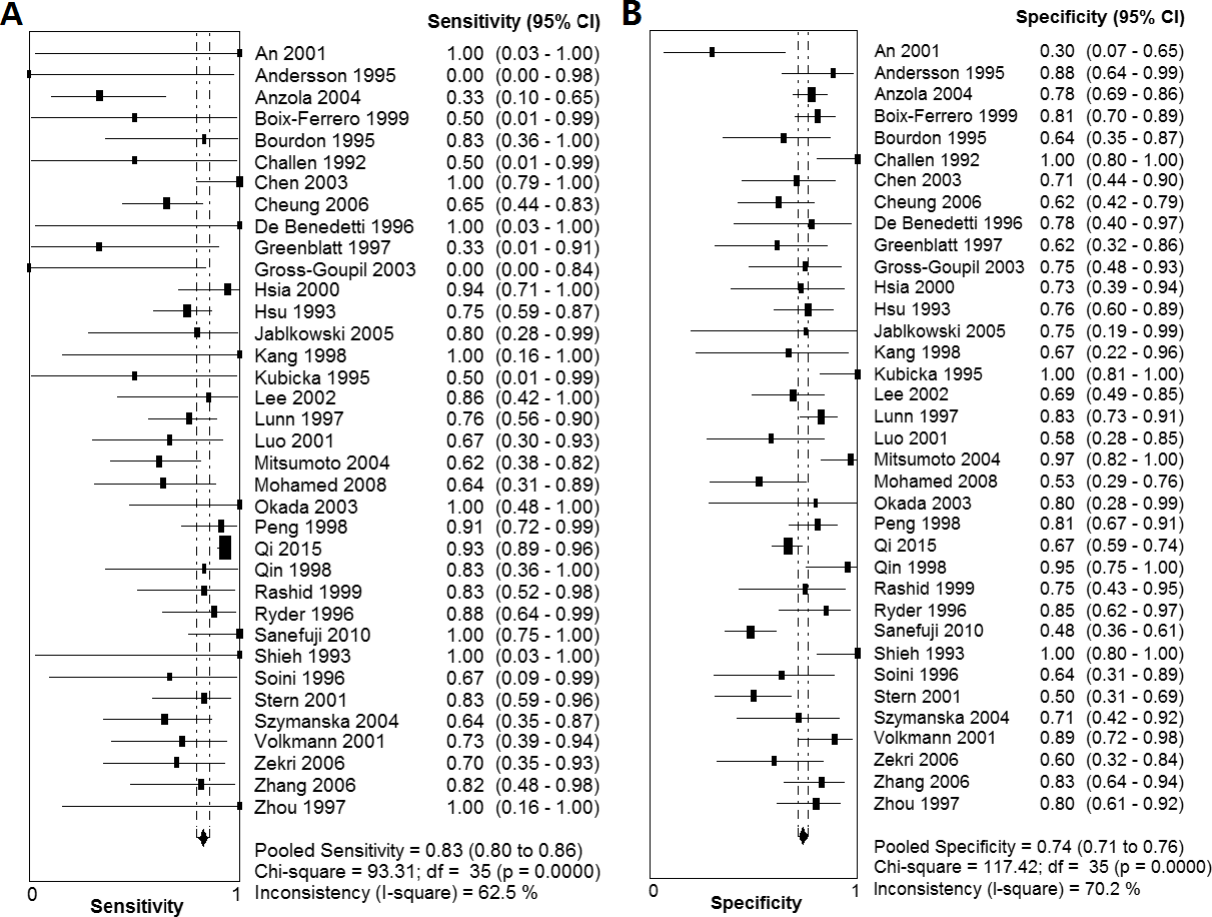
Forest plot of the sensitivity and specificity of IHC-determined p53 overexpression in detecting p53 mutations. (A) Forest plot showing the sensitivity of IHC-determined p53 overexpression in detecting p53 mutations. (B) Forest plot showing the specificity of IHC-determined p53 overexpression in detecting p53 mutations. Abbreviations: CI, confidence interval.

**Fig 3:**
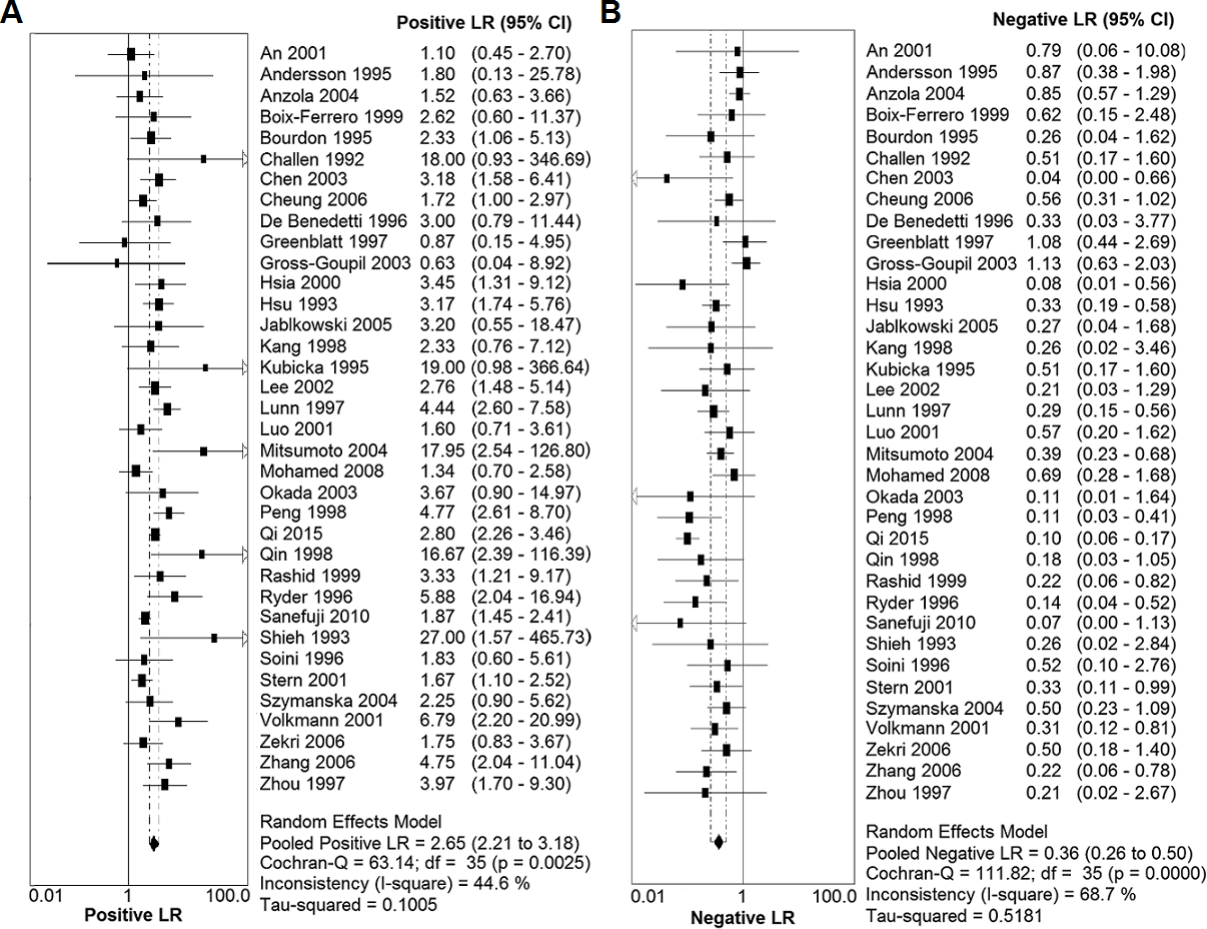
Forest plot of the positive likelihood ratio (PLR) and the negative likelihood ratio (NLR) of IHC-determined p53 overexpression in detecting p53 mutations. (A) Forest plot showing the positive LR of IHC-determined p53 overexpression in detecting p53 mutations. (B) Forest plot showing the negative LR of IHC-determined p53 overexpression in detecting p53 mutations.

**Fig 4:**
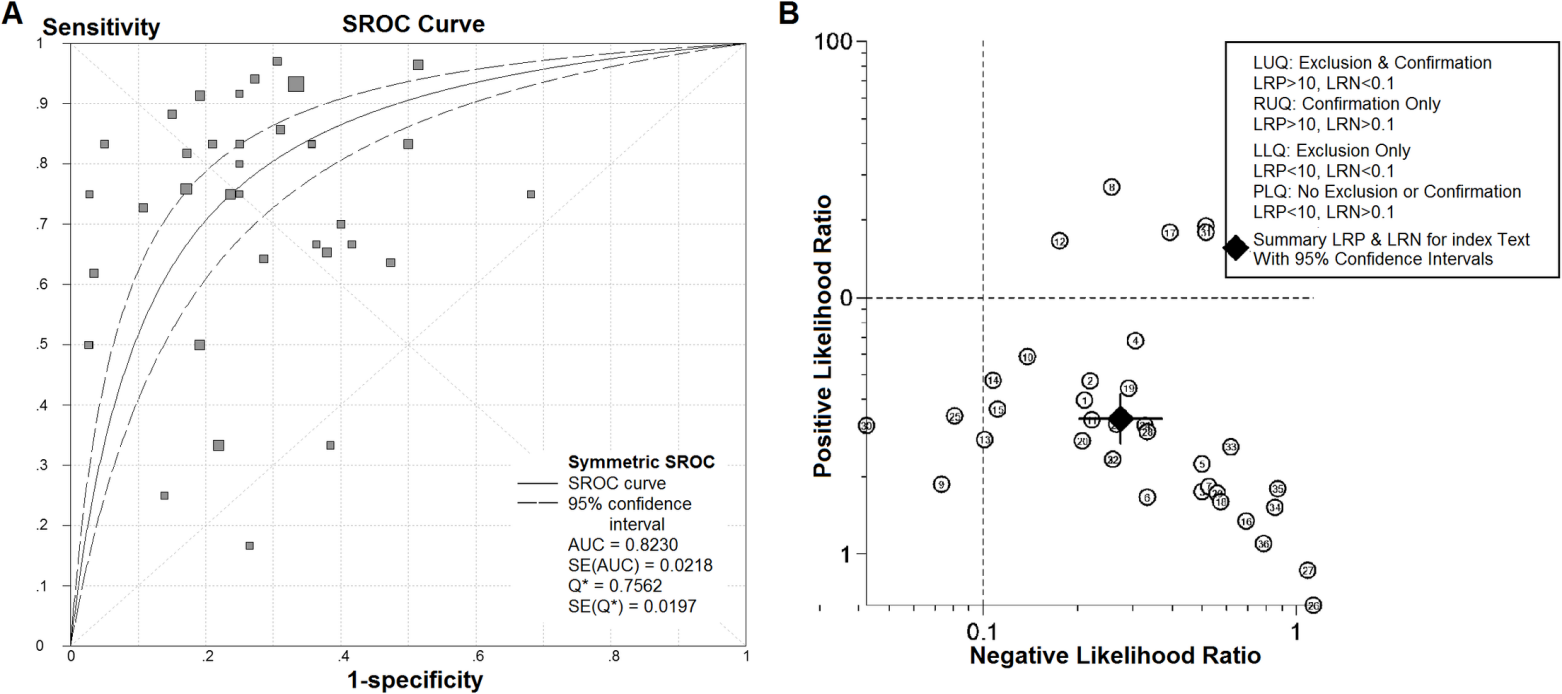
The summary receiver operating characteristic (SROC) curve and the likelihood ratio scattergram for IHC-determined p53 overexpression in the identification of p53 mutations in HCC for all studies. (A) The SROC curve summarizes the overall diagnostic accuracy of IHC-determined p53 overexpression for the identification of p53 mutations. The size of the dots for 1-specificity and sensitivity of the single studies in the ROC space reflects the sample size (number of patients) in the study. (B) The likelihood ratio scattergram shows the diagnostic performance of IHC-determined p53 overexpression in the identification of p53 mutations. Q* = point at which sensitivity and specificity were equal.

As shown in Fig 2, the summary SEN and SPE for IHC-determined p53 overexpression in the diagnostic prediction of p53 mutations in HCC were 0.83 (95% CI: 0.80–0.86) and 0.74 (95% CI: 0.71–0.76), respectively. Moreover, the summary PLR and NLR were 2.65 (95% CI: 2.21–3.18) and 0.36 (95% CI: 0.26–0.50), respectively (Fig 3). The DOR of IHC-determined p53 overexpression in predicting p53 mutations ranged from 0.56 to 105.00 (pooled, 9.77; 95% CI: 6.35–15.02), with significant heterogeneity among the included studies (*I*^2^ = 40.7%, *P* = 0.0067). Additionally, the estimated accuracy and positive and negative predictive values were 77.0%, 63.3% and 88.8%, respectively. The graph of the symmetric SROC curve showed that the AUC of IHC-determined p53 overexpression was 0.8230 (standard error = 0.0218) with a Q-value of 0.7562 (standard error = 0.0197), indicating that IHC-determined p53 overexpression had an overall moderate level of accuracy in the prediction of p53 mutations in HCC (Fig 4A). The likelihood ratio scattergram shows that IHC-determined p53 overexpression has a limited diagnostic ability to identify p53 mutations in HCC (Fig 4B).

### Subgroup analysis

By grouping studies according to the publication year, geographic location, sample size, different IHC antibodies, mutational analysis methods, or prevalence of p53 alterations, subgroup analysis revealed that the diagnostic accuracy of IHC-determined p53 overexpression in identifying p53 mutations in HCC remained consistent (Table 2). Interestingly, the pooled sensitivities were higher in the studies published after the year 2000, as well as in the studies conducted in Asia and Africa or those with a sample size ≥ 46, but the pooled specificities were much lower compared with those of the corresponding subgroups. In the IHC antibodies subgroup analysis, the highest pooled SEN and SPE were from the studies employing the PAb1801 antibody, while the lowest values were from the studies employing the CM-1 antibody. Moreover, for p53 mutational assays, the studies with all cases detected by direct DNA sequencing yielded much higher sensitivities but much lower SPEs, while the group of partial cases that were abnormal in other mutational assays followed by DNA sequencing presented the reverse of these statistics. Furthermore, the pooled SEN was higher in the studies with a high prevalence of p53 alterations (mutation ≥ 35% or overexpression ≥ 46%), but the pooled SPE was lower compared to the subgroup with a low prevalence of p53 alterations.

**Table 2:** The results of subgroup analyses.

Variables	*N*	SEN (95% CI), *I*^2^ (%)	SPE (95% CI), *I*^2^ (%)	PLR (95% CI), *I*^2^ (%)	NLR (95% CI), *I*^2^ (%)	DOR (95% CI), *I*^2^ (%)
**Publication year**						
Before 1999	17	0.79 (0.72−0.85), 0	0.82 (0.78−0.86), 53.1	3.66 (2.88−4.64), 0	0.37 (0.26−0.53), 28.8	13.79 (8.37−22.72), 0
After 2000	19	0.84 (0.81−0.88), 76.1	0.68 (0.65−0.72), 68.0	2.23 (1.80−2.76), 46.8	0.35 (0.22−0.58), 80.1	7.91 (4.18−14.98), 58.6
**Geographic location**						
Asia and Africa	23	0.85 (0.82−0.88), 63.9	0.70 (0.66−0.73), 67.6	2.57 (2.09−3.15), 54.0	0.31 (0.22−0.44), 60.3	10.47 (6.32−17.34), 46.8
Europe and America	13	0.66 (0.53−0.77), 40.5	0.83 (0.78−0.86), 58.2	3.07 (2.02−4.66), 14.8	0.54 (0.349−0.82), 48.8	8.01 (3.50−18.35), 24.5
**Sample size (n, mean)**						
≥ 46	10	0.85 (0.81−0.88), 83.5	0.72 (0.68−0.75), 82.3	2.60 (1.94−3.48), 69.8	0.31 (0.17−0.57), 85.4	10.41 (4.97−21.81), 67.9
< 46	26	0.79 (0.72−0.85), 31.2	0.77 (0.73−0.81), 60.5	2.70 (2.12−3.44), 24.1	0.41 (0.29−0.58), 43.8	9.10 (5.39−15.34), 15.9
**IHC antibodies**						
DO-7 antibody	17	0.73 (0.66−0.79), 58.1	0.71 (0.67−0.75), 69.0	2.27 (1.73−2.99), 47.2	0.46 (0.32−0.67), 61.2	6.48 (3.53−11.88), 39.0
DO-1 antibody	4	0.80 (0.73−0.89), 0	0.84 (0.77−0.89), 0	4.74 (3.21−7.01), 0	0.26 (0.16−0.43), 0	19.75 (8.98−43.42), 0
CM-1 antibody	4	0.71 (0.54−0.85), 17.1	0.59 (0.46−0.71), 0	1.71 (1.21−2.43), 0	0.58 (0.34−0.99), 10.0	3.69 (1.46−9.34), 0
PAb1801 antibody	4	0.80 (0.52−0.96), 0	0.91 (0.82−0.97), 79.4	8.54 (1.82−40.14), 60.3	0.34 (0.15−0.75), 0	30.79 (6.58−144.13), 0
**Mutational assays**						
All DNA sequencing	21	0.89 (0.85−0.92), 57.3	0.70 (0.66−0.74), 75.2	2.43 (1.93−3.06), 39.9	0.32 (0.19−0.55), 73.6	11.10 (5.96−20.68), 36.8
Partial DNA sequencing	15	0.72 (0.65−0.77), 39.4	0.78 (0.74−0.81), 53.4	2.80 (2.09−3.77), 51.5	0.43 (0.31−0.59), 54.5	7.81 (4.32−14.10), 49.3
**Prevalence of p53 alterations**						
Mutation ≥ 35%	15	0.85 (0.82−0.88), 71.2	0.67 (0.64−0.73), 50.0	2.41 (1.92−3.03), 37.0	0.30 (0.20−0.47), 66.7	9.74 (5.18−18.33), 56.4
Mutation < 35%	21	0.76 (0.68−0.83), 48.0	0.77 (0.74−0.80), 75.0	2.94 (2.15−4.04), 54.2	0.43 (0.28−0.66), 61.3	9.91 (5.40−18.18), 25.5
Overexpression ≥ 46%	18	0.87 (0.83−0.90), 63.1	0.64 (0.59−0.68), 32.8	2.21 (1.84−2.65), 33.0	0.29 (0.19−0.45), 60.1	8.82 (4.97−15.67), 47.3
Overexpression < 46%	18	0.71 (0.62−0.78), 42.1	0.83 (0.79−0.86), 58.3	3.71 (2.70−5.11), 27.9	0.46 (0.31−0.68), 62.3	11.21 (5.63−22.29), 36.8

SEN, sensitivity; SPE, specificity; PLR, positive likelihood ratio; NLR, negative likelihood ratio; DOR, diagnostic odds ratio; CI, confidence interval.

### Sensitivity analysis

The leave-one-out method sensitivity analysis showed that the results of the meta-analysis were not impacted by individual studies. Overall, the analytical results showed that the pooled SEN ranged from 0.77 (95% CI: 0.72–0.81, *I*^2^ = 45.7%), by removing Qi et al. [8], to 0.84 (95% CI: 0.81–0.87, *I*^2^ = 56.6%), by removing Anzola et al. [15], and the pooled SPE ranged from 0.73 (95% CI: 0.70–0.76, *I*^2^ = 70.1%), by omitting Lunn et al. [42], to 0.76 (95% CI: 0.73–0.78, *I*^2^ = 64.9%), by omitting Sanefuji et al. [34].

### Publication bias

**Fig 5:**
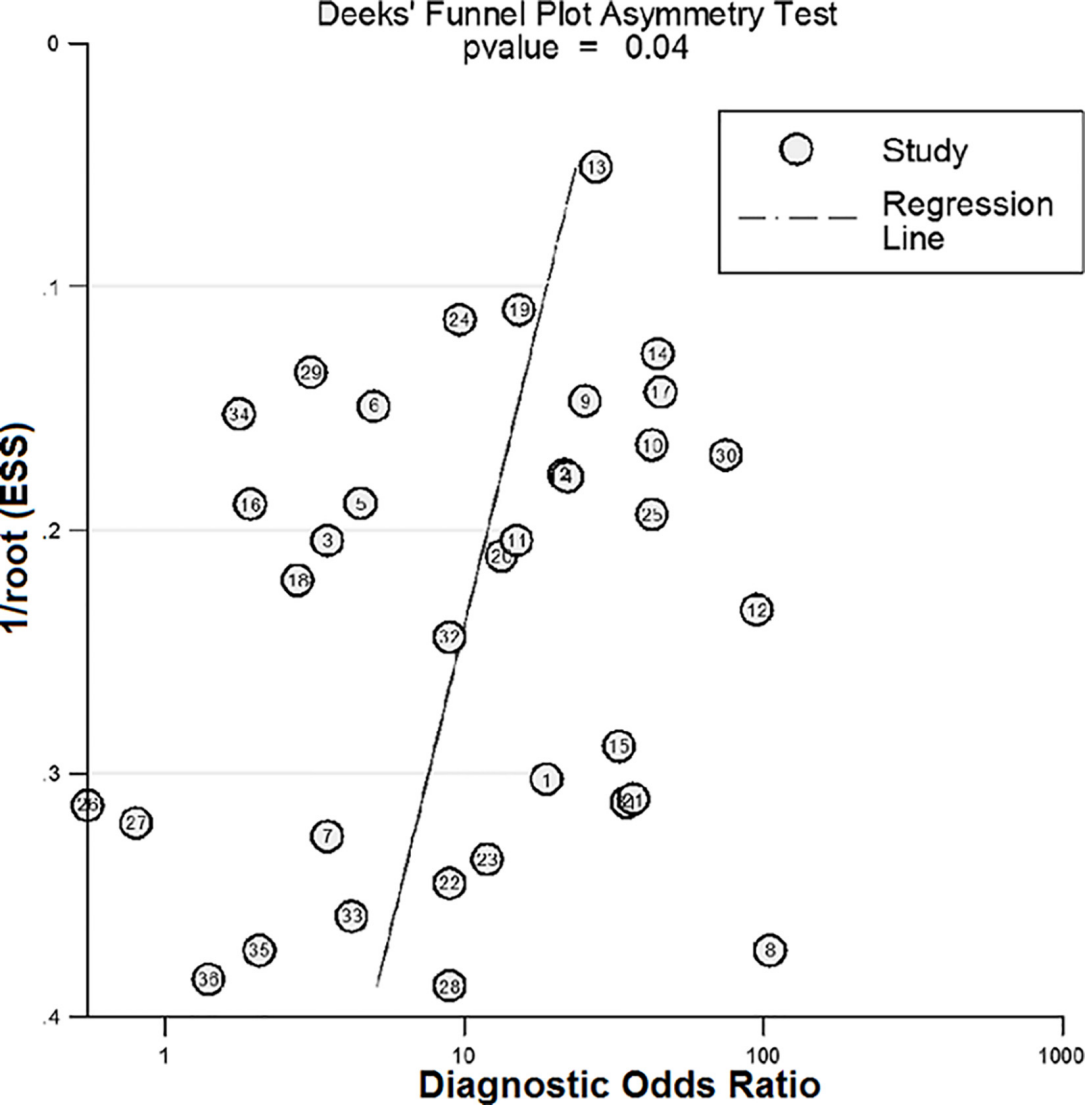
The Deeks’ funnel plot and asymmetry test of the meta-analysis of the 36 included studies.

Fig 5 displays the symmetric shape of the funnel plot. However, the *P* value was less than 0.05 in Deeks’ test, indicating that publication bias may exist in the meta-analysis.

## Discussion

The tumor suppressor gene p53 plays a crucial role in cell cycle control and apoptosis in response to DNA damage, and mutation of the p53 gene has been shown to contribute to carcinogenesis and drug resistance [39,46]. Many studies have reported that p53 mutations are correlated with malignant tumor behaviors in HCC [40,43]. Our previous meta-analysis showed that HCC patients with a mutant p53 gene or p53 protein overexpression had a higher risk of mortality and tumor recurrence than those with wild-type p53 status or low/no p53 expression, which can inform clinical decision-making in HCC [3]. However, it remained unclear whether p53 protein overexpression indicates mutant p53 gene status in HCC. Therefore, the goal of this meta-analysis was to explore the correlation between protein expression and gene mutations of p53 in primary cancer tissues of HCC patients. The results of our meta-analysis, which included 1,659 HCC patients from 36 studies, demonstrated that p53 protein overexpression has a moderate diagnostic concordance to mutational assays in the identification of p53 gene mutations in HCC, with a pooled SEN of 0.83 (95% CI: 0.80–0.86) and SPE of 0.74 (95% CI: 0.71–0.76). Furthermore, the AUC of 0.8230 and the DOR of 9.77 (95% CI: 6.35–15.02) also indicated a moderate overall accuracy.

Usually, wild-type p53 protein is rapidly degraded in a MDM2-dependent manner and is undetectable, while mutant p53 protein can escape from degradation and accumulate to excess levels in the cell nuclei. This p53 protein accumulation has been associated with tumor progression [13,40]; however, studies on p53 protein accumulation have shown inconsistent results. There are several explanations for the differences between the incidence of p53 protein overexpression and p53 genetic alteration: i) other factors, such as the hepatitis virus, may contribute to the transcriptional activation of p53 rather than mutations [5,47]; ii) the presence of a missense mutation [25]; or iii) the threshold values of p53 proteins are different [5,25,30]. Immunoblotting assays revealed that in many tumors, increased p53 was the result of a p53 mutation, but wild-type p53 protein expression was also frequently elevated in HCC. Moreover, elevated wild-type p53 protein expression can upregulate Notch1 (an inhibitor of p53 degradation) in HCC cell lines, resulting in overexpression of wild-type p53 protein [48]. In this meta-analysis, 26.1% (281/1075) of HCC tumor tissues with a wild-type p53 gene exhibited positive staining for p53 protein, while 82.9% (484/584) of specimens with p53 mutations exhibited positive staining. Thus, although the wild-type p53 gene also produced p53 protein upregulation, the association between a p53 mutation and p53 overexpression was easily observable in HCC tissues.

By performing subgroup analysis, we found that the relationship between p53 overexpression and p53 mutations remained unchanged, even when the pooled SENs or SPEs varied due to different stratifications. Notably, the pooled SEN was much higher in high-incidence areas than in low-incidence areas, but the SPE was lower, indicating that in high-incidence areas of HCC, IHC assays for p53 expression accurately predicted p53 alterations with authentic genetic mutations but only showed modest accuracy in identifying wild-type p53 phenotypes with no p53 protein overexpression. However, the pooled SEN and SPE of IHC-determined p53 overexpression in the low-incidence areas showed the opposite results. Specific antibodies for IHC-determined p53 overexpression were critically important in diagnosing p53 mutations. In subgroup analysis, four studies employing IHC PAb1801 antibodies exhibited the best diagnostic performance in identifying p53 mutations compared to the studies using other antibodies, with an SEN of 0.80 (95% CI: 0.52−0.96), SPE of 0.91 (95% CI: 0.82−0.97), and DOR of 30.79 (95% CI: 6.58−144.13), suggesting that the PAb1801 antibody effectively identifies mutant p53 proteins.

In this meta-analysis, significant heterogeneity was observed among the included studies. By excluding each study individually, sensitivity analysis revealed that the diagnostic accuracy of IHC-determined p53 overexpression in identifying p53 mutations in HCC remained consistent. Analytical results showed the lowest pooled SEN (0.77, 95% CI: 0.72–0.81) and the lowest heterogeneity (*I*^2^ = 45.7%) by removing the study by Qi et al. [8], and the greatest pooled SEN (0.84, 95% CI: 0.81–0.87) with significant between-studies heterogeneity (*I*^2^ = 56.6%) by removing the study by Anzola et al. [15]. However, when the two studies were both removed, the between-studies heterogeneity statistic *I*^2^ was reduced to 36.7%, although the effect size remained constant (0.78, 95% CI: 0.73–0.82). In regards to the SPE, by omitting Sanefuji et al. [34], sensitivity analyses yielded the maximal pooled statistics (0.76, 95% CI: 0.73–0.78) and substantial heterogeneity (*I*^2^ = 64.9%, the lowest in the sensitivity analyses of SPE).

Although we quantitatively evaluated the association between IHC-determined p53 overexpression and p53 gene mutations, there were some limitations in our meta-analysis. First, due to the wide time span for the included studies, from 1992 to 2015 (17 studies before 1999), the study design and the process of collecting the data on p53 alterations in HCC patients may vary among these studies, resulting in difficulties in controlling relevant clinical and pathological parameters of the patients and a relatively low study quality. Second, our analysis could not clarify the association between the specific characteristics of p53 mutations and p53 overexpression because individual patient data, such as the mutable sites of p53 in each patient and the exposure to hepatitis B/C virus, AFB1, or other potential mutagens, were lacking. Additionally, there could be a potential language bias in this analysis because only studies written in English, German and Chinese were included. Thus, we suggest that the results of the meta-analysis should be interpreted with caution for the above reasons.

## Conclusion

In summary, our meta-analysis showed that p53 protein overexpression is indeed correlated with p53 gene mutations, suggesting that IHC-determined p53 overexpression has diagnostic concordance to mutational analysis and the identification of p53 gene mutations. This meta-analysis provides quantitative support for the association of IHC-determined p53 overexpression with p53 genetic alterations in HCC patients, especially in high-incidence areas (Asia and Africa). Furthermore, alterations of the tumor suppressor p53 gene were associated with aggressive malignant behaviors and poor patient survival in HCC. Therefore, to obtain a comprehensive account of p53 alterations, simultaneous evaluation of multiple p53 parameters, including p53 protein expression levels and p53 genetic phenotypes, should be performed in future clinical and pathological or prognostic studies and should present compelling evidence of the clinical and prognostic importance of p53 alterations in HCC patients.

## Supporting Information

S1 PRISMA Checklist PRISMA checklist. (DOC)
